# Performance evaluation of the touchscreen-based Muse™ Auto CD4/CD4% single-platform system for CD4 T cell numeration in absolute number and in percentage using blood samples from children and adult patients living in the Central African Republic

**DOI:** 10.1186/s12967-016-1082-7

**Published:** 2016-11-25

**Authors:** Christian Diamant Mossoro-Kpinde, André Kouabosso, Ralph-Sydney Mboumba Bouassa, Jean De Dieu Longo, Edouard Kokanzo, Rosine Féissona, Gérard Grésenguet, Laurent Bélec

**Affiliations:** 1 Laboratoire National de Biologie Clinique et de Santé Publique, Bangui, Central African Republic; 2 Faculté des Sciences de la Santé, Université de Bangui, Bangui, Central African Republic; 3 Centre National de Référence des Infection Sexuellement Transmissibles et de la Thérapie Antirétrovirale, Bangui, Central African Republic; 4 Laboratoire de virologie, Hôpital Européen Georges Pompidou, Paris, France; 5 Université Paris Descartes, Paris Sorbonne Cité, Paris, France; 6 Unité de Recherches et d’Intervention sur les Maladies Sexuellement Transmissibles et le SIDA, Département de Santé Publique, Faculté des Sciences de la Santé de Bangui, Bangui, Central African Republic

**Keywords:** Single-platform, Flow cytometry, Muse™ cell analyzer, CD4 T cell count, Resource-limited settings, Central Africa

## Abstract

**Background:**

The new microcapillary and fluorescence-based EC IVD-qualified Muse™ Auto CD4/CD4% single-platform assay (EMD Millipore Corporation, Merck Life Sciences, KGaA, Darmstadt, Germany) for CD4 T cell numeration in absolute number and in percentage was evaluated using Central African patients’ samples compared against the reference EC IVD-qualified BD FACSCount (Becton–Dickinson, USA) flow cytometer.

**Methods:**

EDTA-blood samples from 124 adults, 10 adolescents, 13 children and 3 infants were tested in parallel at 2 reference laboratories in Bangui.

**Results:**

The Muse™ technique was highly reproducible, with low intra- and inter-run variabilities less than 15%. CD4 T cell counts of Muse™ and BD FACSCount in absolute number and percentage were highly correlated (r^2^ = 0.99 and 0.98, respectively). The mean absolute bias between Muse™ and BD FACSCount cells in absolute number and percentage were −5.91 cells/µl (95% CI −20.90 to 9.08) with limits of agreement from −77.50 to 202.40 cells/µl, and +1.69 %CD4 (95% CI ±1.29 to +2.09), respectively. The percentages of outliers outside the limits of agreement were nearly similar in absolute number (8%) and percentage (10%). CD4 T cell counting by Muse™ allowed identifying the majority of individuals with CD4 T cell <200, <350 or <750 cells/µl corresponding to the relevant thresholds of therapeutic care, with sensitivities of 95.5–100% and specificities of 83.9–100%.

**Conclusions:**

The Muse™ Auto CD4/CD4% Assay analyzer is a reliable alternative flow cytometer for CD4 T lymphocyte enumeration to be used in routine immunological monitoring according to World Health Organization recommendations in HIV-infected adults as well as children living in resource-constrained settings.

## Background

In 2013, the World Health Organization (WHO) published the first consolidated guidelines on the use of antiretroviral treatment (ART) for HIV treatment and prevention across all age groups and populations [1]. The 2013-revised WHO guidelines emphasized the need for laboratory monitoring, firstly based on immunological assessment of CD4 T lymphocytes numeration to start ART and monitor treated patients and secondly based on HIV-1 RNA load, in order to monitor treatment efficacy, early therapeutic failure and therapeutic switch in patients on ART [2–5]. More recently, the ambitious UNAIDS Fast-Track targets for 2020, refers to achieving major reductions in HIV-related mortality and new HIV infections and the 90–90–90 targets, pushed countries to further accelerate their HIV responses in the coming years. A comprehensive revision of the WHO guidelines on the use of ART has been undertaken in 2015 and based on new scientific evidence and lessons from antiretroviral programs implementation [6] and recently consolidated in 2016 [7]. Basically, the key recommendation that was developed during the WHO revision process in 2015 and 2016 is that ART should be initiated in everyone living with HIV at any CD4 T cell count [6, 7]. Indeed, earlier use of ART results in better clinical outcomes for people living with HIV compared with delayed treatment. The HIV-1 RNA load remains the principal biological marker to monitor ART efficacy and early therapeutic failure [6, 7]. However, CD4 T cell counting remains currently an important biological marker for ART monitoring for at least 4 major reasons: (1) The marker is well known to physicians and largely implemented throughout Africa; (2) WHO thresholds for ART initiation as a priority remains based on CD4 T cell enumeration while ART is not universally available; (3) Immunological failure may be important to diagnose in order to confirm therapeutic failure by targeted HIV-1 viral load when HIV load is not universally implemented; and finally (4) CD4 T cell levels are important to determine the prophylaxis of opportunistic infections. Finally, CD4 T cell counting will remain an important biological marker for ART during the next few years.

A number of technologies are available for CD4 T cell enumeration, with considerable variation in cost, complexity and operating requirements [8–10]. The traditional approach to measure absolute CD4 T lymphocyte counts is to use the total leukocyte count (or lymphocyte count) obtained from the hematology analyzer and then use the percentage of CD4 T lymphocytes from the flow cytometric analysis to calculate the absolute values—the so-called “dual platform” (DP) approach, which is however often associated with inter-laboratory variation as high as 40% [9, 10]. Thus, the need to derive accurate and precise absolute CD4 T lymphocyte counts has led to the development of instruments that can produce both percentage and absolute values, termed “single-platform” approach. Affordable CD4 T cell counting has gradually become possible by using simple, compact, robust and low-cost new generation cytometers operating as single-platform volumetric instruments without the use of expensive microbeads [9, 11–20]. The recently developed Muse™ Auto CD4/CD4% system (EMD Millipore Corporation, Merck Life Sciences, KGaA, Darmstadt, Germany) consists of a compact, portable and easy-to-use cell analyzer, software and optimized reagents.

Finally, the aim of the present study was to evaluate the usefulness of the simplified Muse™ Auto CD4/CD4% system for CD4 T cell numeration in absolute count and in percentage, compared against reference flow cytometry method. Because it is critical that country programs consider whether CD4 analyzers can give accurate and reproducible results, as well as being appropriate for the setting, we focused our field evaluation on bias and misclassification probabilities of different CD4 T cell thresholds that are important for ART initiation as a priority according to WHO guidelines.

## Methods

### Clinical specimens and processing

In June 2016, tri-potassium ethylenediaminetetraacetic acid (K_3_-EDTA)-blood samples obtained by venipuncture in Vacutainer tubes (Becton–Dickinson, Franklin Lakes, NJ, USA) were received from HIV-1-infected patients followed for routine biological monitoring. The monitoring was conducted at the *Centre National de Référence des Infections Sexuellement Transmissibles et de la Thérapie Antirétrovirale* («*CNRISTTAR*»), Bangui, Central African Republic, which is a research laboratory devoted to HIV screening and monitoring, affiliated to University of Bangui. HIV-1-infected children attending the *Complexe Pédiatrique* of Bangui for their antiretroviral treatment follow up were also included. Two aliquots were kept at ambient temperature. No extra specimens were required. All blood samples were anonymous, unlinked to identifiers and no mention of possible antiretroviral treatment could be made. Each aliquot was first subjected to CD4 T cell count by Muse™ Auto CD4/CD4% system, within 1 h, at the *CNRISTTAR*. The second aliquot was used in parallel, within 1 h, at the *Laboratoire National de Biologie Clinique et de Santé Publique* of Bangui, The HIV National Reference Laboratory, for measurement on BD FACSCount (Becton–Dickinson), a dedicated reference clinical instrument for CD4 T cell counting [9], chosen as flow cytometry reference analyzer.

### CD4 T cell measurements

CD4 T cell counting was performed in parallel on 2 different systems: (1) the BD FACSCount; and (2) the Muse™ Auto CD4/CD4% system. Both the BD FACSCount and the Muse™ Auto CD4/CD4% systems are based on the flow cytometry principle, using BD FACSCount and Muse™ softwares enable automated identification of the lymphocyte populations of interest, and calculation of the CD4 T cells in absolute counts and percentages.

The BD FACSCount consists of a single-platform benchtop, a modified flow cytometer, using a standard reagent kit [9]. The instrument is equipped with a green laser for excitation of 2 fluorescent parameters: phycoerythrin (PE) and tandem fluorochrome composed of PE and indodicarbocyanine (Cy™5) (PE-Cy™5). Recently, new reagents and software have been updated to allow the additional measurements of CD4% essential for monitoring paediatric patients in addition to absolute CD4 T cell [21–23]. In this study, the new FACSCount CD4 reagent kit was used, consisting of a single tube containing a mixture of 3 monoclonal antibodies, CD4/CD14/CD15 (conjugated with PE/PE-Cy™5/PE-Cy™5, respectively), a nuclear DNA fluorescent dye and a known number of fluorescent microbeads. Monoclonal antibody to CD14 recognizes a human monocyte/macrophage antigen, whereas monoclonal antibody to CD15 recognizes a human myelomonocytic antigen.

The Muse™ Auto CD4/CD4% system consists of a compact, portable and easy-to-use cell analyzer, software and optimized reagents. The Muse™ analyser uses novel, miniaturized optics and microcapillary laser technology. In brief, a sample of fluorescent labeled cells is aspirated into a uniquely proportioned microcapillary flow cell. A green laser excites the cells and each cell emits a signal that is individually detected by photomultipliers and a photodiode. The Muse™ Auto CD4/CD4% kit is intended to be performed on a Muse™ Cell Analyzer with Muse™ Auto CD4% Software, which includes 3 modules: Muse™ Auto CD4/CD4%, System Check and Complete System Clean. The Muse™ Auto CD4/CD4% kit is intended to identify and quantify both absolute CD4 T cell counts and CD4% values in whole blood samples. The kit consists of the Muse™ Auto CD4/CD4% antibody cocktail, a proprietary mixture of antihuman lymphocyte antibodies and a monoclonal anti-human CD4 antibody. The kit also contains Muse™ 1× Lysing Solution to lyse erythrocytes. The anti-human lymphocyte antibodies detect all human lymphocytes. The CD4 antibody identifies human CD4-positive helper/inducer T cells (HLA Class II reactive) and recognizes a 60-kDa surface antigen. Monocytes also express CD4 but at lower density and have no co-expression of the other lymphocyte markers detected by reagents in this kit. The CD4% values are the absolute counts of the CD4 T helper cells expressed as a percentage of total lymphocytes in EDTA whole blood samples from adult and pediatric donors. All relevant data and results are immediately available.

To ensure quality control of the Muse™ Auto CD4/CD4% system and the reference flow cytometric BD FACSCount with regard to the performances of the analyzers, the same lots of reagents were used throughout the study. To assess the accuracy of the reference BD FACSCount system, internal quality control reagents consisting of 3 known concentrations of fluorochrome-integrated beads [low (50 beads/µl), medium (250 beads/µl) and high (1000 beads/µl)] were analyzed prior to measuring each batch of stained whole blood samples. Systematic contractual maintenance of the BD FASCCount analyzer was carried out each year by Becton–Dickinson, Douala, Cameroon. Finally, external quality control of the reference BD FACSCount platform at *Laboratoire National de Biologie Clinique et de Santé Publique* of Bangui was organized each year by the medical biology laboratory of hôpital Européen Georges Pompidou, Paris, France.

### Assessment of the precision of CD4 T cell counting by Muse™ Auto CD4/CD4

The precisions of CD4 T cell counting on the Muse™ Auto CD4/CD4% system, including intra-assay (as an evaluation of the repeatability) and inter-assay (as an evaluation of the reproducibility) variations, were assessed using 3 different EDTA-blood samples stored at room temperature with CD4 T cell counts of clinical interest (<200; 200–350; >750 cells/µl). The intra-assay variation, which assesses the tube-to-tube variability, including errors due to operator, was determined by repeating the entire CD4 staining procedure 10 times on the 3 blood samples. Inter-assay variation (day-to-day) takes into account the variations of pipetting made by the technician (tube-to-tube variability). This was assessed by repeating the entire procedure 10 times on 3 separate days, including pipetting, sample preparation, staining and sample acquisition, in the 3 pools. Interperson variation (between different technicians) was not assessed. Precision was expressed as the coefficient of variance (CV) obtained by dividing the standard deviation (SD) of all measurements by their mean (CV% = SD × 100/mean) [24].

### Statistical analyses

The following definitions for adults, adolescents, children and infants were used according to the 2015-revised WHO recommendations [6]: an adult is a person older than 19 years, an adolescent is a person 10–19 years old inclusive, a child is a person younger than 10 years and an infant is a child younger than 1 year of age.

The Method Validator software, version 1.1.9.0. (Philippe Marquis, France) and the SAS-PC software (version 8.2, SAS Institute, Cary, NC, USA) were used for statistical analyses. Firstly, correlations between the absolute and percentage CD4 T cell counts obtained by the reference BD FACSCount system and the Muse™ Auto CD4/CD4% system were established by the unweighted linear regression as well as the Passing-Bablok nonparametric method which is fairly less sensitive to outliers [25]. The level of significance for correlation tests was set at α < 0.05. Secondly, the agreement between BD FACSCount system and Muse™ Auto CD4/CD4% system was depicted by different plots as proposed by Bland and Altman [26, 27]. The Bland–Altman analysis examines, in a discriminative fashion, whether the methods agree sufficiently well to be used interchangeably. The average of values obtained by the 2 methods is displayed on the x axis and plotted against the difference between the 2 methods shown on the y axis. The average difference between the 2 methods, referred to as bias, was marked on the graph by a horizontal line and the limits of agreement with a 95% confidence interval (CI) were also depicted.

The sensitivities and specificities of the Muse™ Auto CD4/CD4% system to diagnose patients according to different relevant WHO thresholds of therapeutic care were evaluated in the field. Thus, the threshold of 200 cells/µl corresponds to the threshold of immune-restauration under ART and the threshold for therapeutic initiation according to the 2006-revised WHO recommendations [28]. The threshold of 350 cells/µl corresponds to the WHO threshold for antiretroviral treatment initiation in adults and children aged more than 5 years according to the 2010-revised WHO guidelines [29] and the threshold for antiretroviral treatment initiation as a priority for adults, adolescents (10–19 years old) and children aged more than 5 years according to the 2015-revised WHO guidelines [6] and to the 2016-consolidated WHO guidelines [7]. Finally, the threshold of 750 CD4 T cells/µl and 25 %CD4+ correspond to the absolute and percent CD4 T cell count 2010-revised WHO thresholds for antiretroviral treatment initiation in children aged between 24 and 59 months [29] and the thresholds for antiretroviral treatment initiation as a priority for children aged more than 2 years and less than 5 years [6, 7].

For clinical significance of the measurement differences on treatment decision, the Cohen’s κ coefficient was calculated on study population (http://faculty.vassar.edu/lowry/kappa.html) [30].

### Ethics statement

We used the excess of routine blood samples from HIV-infected patients attending the *CNRISTTAR* or the *Complexe Pédiatrique* for routine CD4 T cell enumeration. Blood sample records were made anonymous and identified by giving them a consecutive study code prior to analysis. The study was formally approved by the Scientific Committee of *Faculté des Sciences de la Santé* (“FACSS”) of Bangui, (so-called “*Comité Scientifique de la Validation des Protocoles et des Résultats de Recherche en Santé*”/“CSVPR”) constituting the National Ethical Committee (agreement #2UB/FACSS/CSVPR). Finally, the return of laboratory results to clinicians was conducted to achieve a better management of the treated patient. Feedback was given to the children’s parents and their pediatricians on all tested parameters carried out during the study period, allowing changes of antiretroviral treatment and improvement of medical care. All participants, including children’s guardians or parents, signed an informed consent. For the purpose of this independent study, the Muse™ Auto CD4/CD4% and FACSCount CD4 reagents were purchased. Furthermore, EMD Millipore Corporation was never involved in running and/or analyzing the samples and/or writing the present paper.

## Results

### Precision of CD4 T cell measurements by Muse™ Auto CD4/CD4% system

The intra-assay and inter-assay CVs were tested using 3 blood samples in total, one for each range of low (<200 cells/µl), middle (between 200 and 350 cells/µl) and high (>750 cells/µl) CD4 T cell counts (e.g. within the ranges of values of medical interests). The results are shown in Table 1.

**Table 1:** Intra-assay (as an estimation of the repeatability) and inter-assay (as an estimation of the reproducibility) precisions of single-platform Muse™ Auto CD4/CD4% system for CD4 T cells measurement, expressed in absolute number and in percentage

Range	CD4 T cells in absolute number				CD4 T cells in percentage			
	Samples* (intrarun assay)	Intra-assay CV**** (%)	Samples** (interrun assay)	Inter-assay CV (%)	Samples* (intrarun assay)	Intra-assay CV***** (%)	Samples** (interrun assay)	Inter-assay CV (%)
<200 cells/µl	161.5 ± 14.9	9.2	199.7 ± 20.8	10.4	19.0 ± 0.8	4.3	16.0 ± 1.8	11.0
200–350 cells/µl	266.1 ± 15.1	5.7	252.8 ± 33.5	13.2	16.6 ± 0.8	5.1	13.7 ± 1.8	13.7
>750 cells/µl	1856.9 ± 194.7	10.5	723.6 ± 56.6	7.8	28.3 ± 1.0	3.7	36.2 ± 3.2	9.0
	Mean intra-assay CV***	8.5	Mean inter-assay CV***	10.4	Mean intra-assay CV***	4.4	Mean inter-assay CV***	11.2

* Mean ± standard deviation of results obtained by tenfold repeating the *same day* acquisition on Muse™ Auto CD4/CD4% system of the blood sample of each range of CD4 T cells

** Mean ± standard deviation of results obtained by tenfold repeating on *3 separate days* acquisition on Muse™ Auto CD4/CD4% system of the blood sample of each range of CD4 T cells

*** Precision is the coefficient of variance (CV) obtained by dividing the standard deviation (SD) of all the intrarun or interrun measurements by their mean (CV% = SD × 100/mean); the mean CV is the mean of the 3 CVs calculated at 3 different counts of blood CD4 T cells

**** Intrarun CVs obtained with BD FACSCount (Becton–Dickinson, Franklin Lakes, NJ, USA) previously estimated in Dar es Salam (Tanzania) was 6.8% for CD4 T cell counts expressed in absolute number and 5.0% for CD4 T cell counts in percentage [41]

***** Interrun CVs obtained with BD FACSCount (Becton–Dickinson, Franklin Lakes, NJ, USA) estimated in Dar es Salam (Tanzania) were 8.0% for low CD4 T cell counts expressed in absolute number and 7.9% for low CD4 T cell counts in percentage; and 7.0% for high CD4 T cell counts expressed in absolute number and 5.6% for high CD4 T cell counts in percentage [41]

For CD4 T cell expressed in absolute number, the mean intra-assay CVs of Muse™ Auto CD4/CD4% system was 8.5%; the inter-assay CVs of the analyzer appeared slightly higher than intrarun CVs for CD4 T cell counts below 350 cells/µl, with a mean interrun variability of 10.4%.

For CD4 T cell expressed in percentage, the mean intra-assay CVs of Muse™ Auto CD4/CD4% system was 4.4%; the inter-assay CVs of the analyzer appeared higher than intra-assay CVs for all CD4 T cell counts, with a mean interrun variability of 11.2%.

Thus, the repeatability appeared generally higher than the reproducibility for CD4 T cell measurements expressed in absolute number or in percentage. Furthermore, the reproducibility of CD4 T cell measurement in percentage was slightly higher than the reproducibility of CD4 T cell measurement in absolute number, at each level of CD4 T cell. Nevertheless, the repeatabilities and reproducibilities were always below 15% whatever the CD4 T cell levels and the CD4 T cell expression (absolute number or percentage).

### Accuracy of CD4 T cell measurements by Muse™ Auto CD4/CD4% system

Parallel CD4 T cell measurements on both instruments, the Muse™ Auto CD4/CD4% system and BD FACSCount system, were done for a total of 150 tested blood samples, obtained from study patients (median age of 36 years; range 0.7–66 years; 106 females), including 124 adults (over 19 years), 10 adolescents (10–19 years old), 13 children (from 1 to 9 years) and 3 infants (under 1 year).

**Fig. 1:**
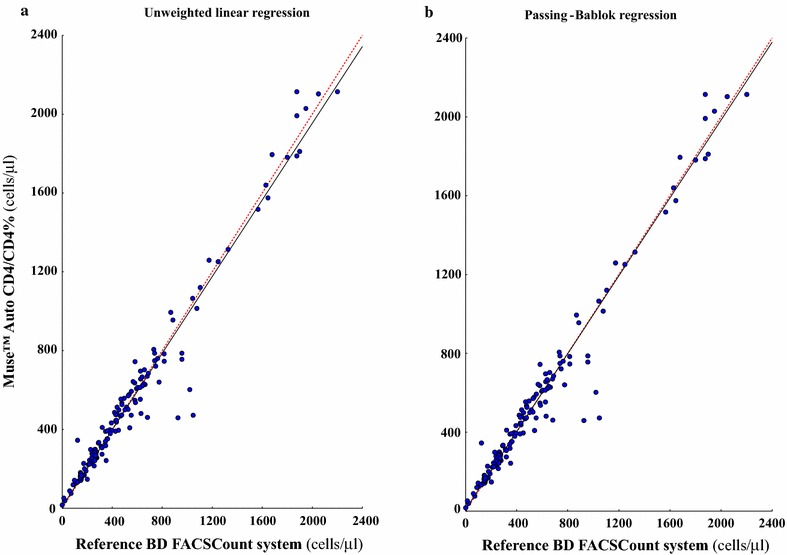
Unweighted linear regression (**a**) and Passing-Bablok regression (**b**) tests between CD4 T cell count measurements in 150 HIV-1-infected patients [including 124 adults (>19 years), 10 adolescents (10–19 years old), 13 children (>1 to 9 years) and 3 infants (<1 year)], expressed in absolute number (cells/µl) obtained in parallel by the Muse™ Auto CD4/CD4% system and by the reference BD FACSCount system. By unweighted linear regression, slope and Y-intercept values for the resultant trend line were 0.973 (95% CI, [+0.941 to +1.005]) and 9.0 (95% CI, [−14.0 to +31.9]), respectively. By Passing-Bablok regression, slope and Y-intercept values for the resultant trend line were 0.988 (95% CI, [+0.962 to +1.014]) and 8.1 (95% CI, [−1.8 to +16.2]), respectively. The *diagonal dotted lines* represent the ideal lines (no bias). The *full lines* represent the regression lines of the distribution

**Fig. 2:**
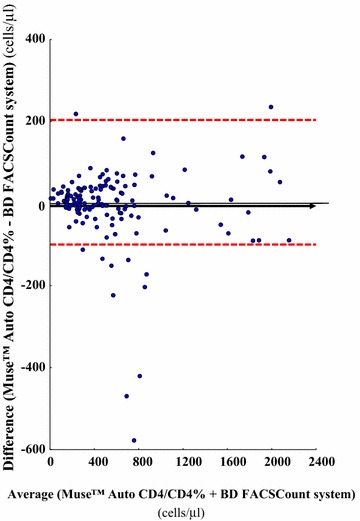
Bland–Altman analyses on the relative differences between the CD4 T cell counts in 150 HIV-1-infected patients obtained by Muse™ Auto CD4/CD4% system and BD FACSCount system compared with the average CD4 T cell count in absolute number (cells/µl). The *full line* represents the mean relative difference and the *dotted lines* represent the superior and inferior limits of agreement. The *arrow* corresponds to the x *abscise* axis

**Fig. 3:**
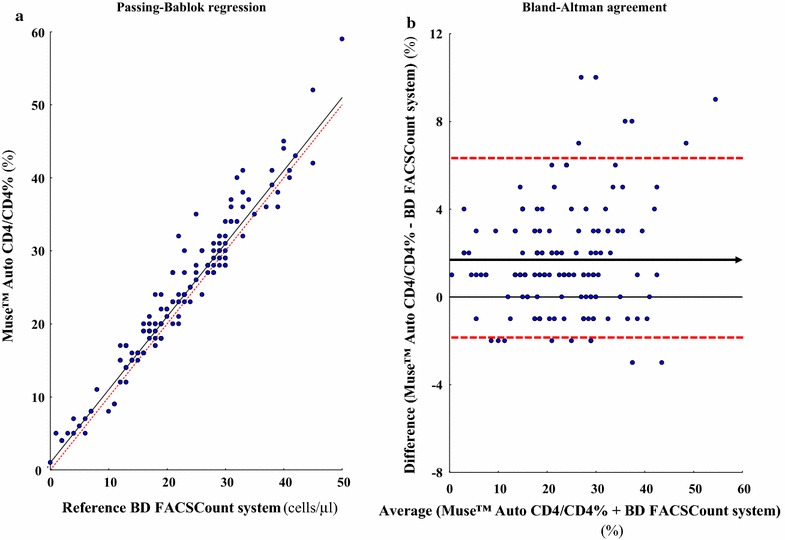
**a** Passing-Bablok regression between CD4 T cell count measurements in 150 HIV-1-infected patients expressed in percentage obtained in parallel by the Muse™ Auto CD4/CD4% system and by the reference BD FACSCount system. Slope and Y-intercept values for the resultant trend line were 1.00 (95% CI [0.999–1.009]) and +1.0 (95% CI [+0.2 to +1.0]), respectively. The *diagonal dotted lines* represent the ideal lines (no bias). The *full lines* represent the regression lines of the distribution. **b** Bland-Altman analyses on the relative differences between the CD4 T cell counts obtained by Muse™ Auto CD4/CD4% system and BD FACSCount system compared with the average CD4 T cell count in percentage. The *full line* represents the mean relative difference and the *dotted lines* represent the superior and inferior limits of agreement. The *arrow* corresponds to the x *abscise* axis

The accuracy of CD4 T cell measurements by the Muse™ Auto CD4/CD4% system was carried out against the results obtained by the BD FACSCount system chosen as reference for CD4 T cell counting. The absolute bias (and the limits of agreement) for CD4 T cell counting in absolute count and percentage are shown in Tables 2 and 3 for the study in adults and children, by the Muse™ Auto CD4/CD4% system and the BD FACSCount system. In addition, the Fig. 1 depicts the unweighted linear and the Passing-Bablok regressions; the Fig. 2 shows the Bland–Altman analyses between the CD4 T cell count results obtained by Muse™ Auto CD4/CD4% system and BD FACSCount flow cytometer, in absolute number, in the 140 study HIV-1-infected adults, adolescents and children more than 5 years and 10 HIV-1-infected children and infants less than 5 years; and the Fig. 3 depicts the Passing-Bablok regression and the Bland–Altman agreement when the CD4 T cell count obtained from the same population are expressed in percentage.

**Table 2:** CD4 T cell counting in absolute count and percentage in 150 HIV-1-infected patients living in Central African Republic, including 124 adults, 10 adolescents, 13 children and 3 infants, by the single-platform Muse™ Auto CD4/CD4% system and by the BD FACSCount system

Categories	Adults, adolescents and children ≥ 5 years	Children < 5 years and infants
Number	140	10
Absolute CD4 T cells (cells/µl)		
Muse™ Auto CD4/CD4% system		
Mean ± SD	1003 ± 1397	1263 ± 1081
BD FACSCount system		
Mean ± SD	940 ± 1325	1203 ± 1056
Absolute bias (limits of agreement)^a^		
Mean (±1.96 SD)	+62.5 (−77.4; +202.4)	+60.0 (10.1; +109.8)
Percent CD4 T cells (%CD4)		
Muse™ Auto CD4/CD4% system		
Mean ± SD	15.5 ± 20.5	17.5 ± 14.8
BD FACSCount system		
Mean ± SD	13.0 ± 18.4	18.0 ± 16.9
Absolute bias (limits of agreement)^a^		
Mean (±1.96 SD)	+2.5 (−1.6; +6.7)	−0.5 (−4.6; +3.6)

*SD* standard deviation

^a^The Bland–Altman analysis was carried out to calculate the absolute bias and limits of agreement which are the 95% confidence intervals (±1.96 × SD) of the mean bias of all paired measurements in a given category (27)

**Table 3:** CD4 T cell counting in absolute count and percentage in 150 HIV-1-infected patients living in Central African Republic, by the single-platform Muse™ Auto CD4/CD4% system and by the BD FACSCount system, at various CD4 T cell count ranges according to the BD FACSCount system results

Categories	<200 cells/µl	200–350 cells/µl	>350 cells/µl
Number	30	35	85
Absolute CD4 T cells (cells/µl)			
Muse™ Auto CD4/CD4% system			
Mean ± SD	90 ± 106	276 ± 13	1225 ± 1082
BD FACSCount system			
Mean ± SD	79 ± 107	273 ± 4	1281 ± 844
Absolute bias (limits of agreement)^a^			
Mean (±1.96 SD)	+9.5 (+2.6; +16.4)	+11.5 (+4.6; +18.4)	−73.0 (−491.5; +345.1)
Percent CD4 T cells (%CD4)			
Muse™ Auto CD4/CD4% system			
Mean ± SD	10.0 ± 12.7	20.0 ± 5.7	32.0 ± 2.8
BD FACSCount system			
Mean ± SD	8.5 ± 12.0	16.0 ± 2.8	28.0 ± 2.9
Absolute bias (limits of agreement)^a^			
Mean (±1.96 SD)	+0.5 (−0.8; +1.9)	+3.0 (−5.3; +11.2)	+1.0 (−7.3; +9.3)

*SD* standard deviation

^a^The Bland–Altman analysis was carried out to calculate the absolute bias and limits of agreement which are the 95% confidence intervals (±1.96 × SD) of the mean bias of all paired measurements in a given category [27]

Mean ± SD of CD4 T cell/µl expressed in absolute number was 1003 ± 1397 cells/µl (range 15–2113) by Muse™ Auto CD4/CD4% system and 940 ± 1325 cells/µl (range 16–2203) by BD FACSCount flow cytometer (P > 0.5). The unweighted linear regression analysis on all 150 available T cell results expressed in absolute count revealed a high correlation between CD4 T cell counts obtained by Muse™ Auto CD4/CD4% system and BD FACSCount flow cytometer (Fig. 1a). Similarly, the non-parametric Passing-Bablok regression analysis showed high correlation between Muse™ Auto CD4/CD4% system and BD FACSCount flow cytomete (r^2^ = 0.99) with a slope of 0.98 (95% IC 0.96–1.00) and an intercept of +8.1 (95% CI −1.8 to 16.2) [<200 cells/ml: r^2^ = 0.87, slope = 0.86, intercept = +33.1; 200–350 cells/ml: r^2^ = 0.97, slope = 0.95, intercept = +3.1; >350 cells/ml: r^2^ = 0.98, slope = 0.98, intercept = −7.7] (Fig. 1b). The relation between Muse™ Auto CD4/CD4% system and BD FACSCount flow cytometer did not differ from linearity (P > 0.4). The mean absolute bias measured by Bland–Altman analysis between CD4 T cell/µl obtained by Muse™ Auto CD4/CD4% system and BD FACSCount flow cytometer over the entire range of CD4 T cell results, were −5.91 cells/µl (95% CI −20.90 to 9.08) with limits of agreement from −77.50 to 202.40 cells/µl (Fig. 2).

Analysis of CD4 T cell count measurement expressed in percentage showed a high correlation and a close agreement between both CD4 T cell counting methods, similarly to CD4 T cell count expressed in absolute numbers. Mean ± SD CD4 T cell count in percentage was 15.5 ± 20.5 %CD4+ (range 1–59) by Muse™ Auto CD4/CD4% system and 13.0 ± 18.4 %CD4+ (range 0–50) by BD FACSCount flow cytometer (P > 0.5). Results of CD4 T cell count in percentage by Muse™ Auto CD4/CD4% system and BD FACSCount flow cytometer were highly correlated by non-parametric Passing-Bablok regression analysis (r^2^ = 0.98) with a slope of 1.00 (95% IC 0.97–1.05) and an intercept of +1.4 (95% CI +0.4 to +2.3) [<200 cells/ml: r^2^ = 0.99, slope = 1.02, intercept = +1.4; 200–350 cells/ml: r^2^ = 0.96, slope = 0.91, intercept = +2.7; >350 cells/ml: r^2^ = 0.97, slope = 1.03, intercept = +0.9] (Fig. 3a), as well as unweighted linear regression analysis (not shown). The relation between Muse™ Auto CD4/CD4% and BD FACSCount did not differ from linearity (P > 0.5). The mean of absolute bias between percentage of CD4 T cell obtained by Muse™ Auto CD4/CD4% system and BD FACSCount flow cytometer was +1.69 %CD4 (95% CI ±1.29 to +2.09) with limits of agreement from −1.65 to +6.67 %CD4.

The numbers of outliers outside the limits of agreement were similar when the CD4 T cell count was expressed in absolute count (12/150 = 8%) as when it was given in percentage (15/150 = 10%) (P > 0.5 by Student *t* test) (Figs. 2, 3).

Finally, the Bland–Altman analysis on the relative differences between the absolute and percent CD4 T cell counts obtained with Muse™ Auto CD4/CD4% system and BD FACSCount system with the average absolute CD4 T cell counts results showed a close agreement between both methods.

### Identification of clinically-relevant thresholds by Muse™ Auto CD4/CD4% system

The sensitivity and specificity of CD4 T cell counting in absolute number on the Muse™ Auto CD4/CD4% system to identify relevant thresholds of CD4 T cell count according to the WHO recommendations are depicted in Table 4.

**Table 4:** Sensitivity and specificity of CD4 T cell counting by the Muse™ Auto CD4/CD4% system to identify patients having less than (or more than) 200, 350, 750 CD4 T cells/µl and 25 %CD4+, calculated on the 150 available CD4 T cell count measurements, including the reference results obtained by BD FACSCount

	Sensitivity^a^ (%)	Specificity^a^ (%)	Cohen’s κ coefficient^$^
Thresholds			
200 CD4 T cells/μl*	96.7	100	0.98
350 CD4 T cells/μl**	100	100	1.00
750 CD4 T cells/μl***	100	83.9	0.89
25 %CD4+***	95.5	100	0.95

* 200 CD4 T cells/µl: threshold of immune-restauration under antiretroviral treatment and the threshold for therapeutic initiation according to the 2006-revised WHO recommendations [28]

** 350 CD4 T cells/µl: WHO threshold for antiretroviral treatment initiation in adults and children aged more than 5 years according to the 2010-revised WHO guidelines [29] and threshold for antiretroviral treatment initiation as a priority for adults, adolescents (10–19 years old) and children aged more than 5 years according to the 2015-revised WHO guidelines [6] and to the 2016-consolidated WHO guidelines [7]

*** 750 CD4 T cells/µl and 25 %CD4+: absolute and percent CD4 T cell count 2010-revised WHO thresholds for antiretroviral treatment initiation in children aged between 24 and 59 months [29] and thresholds for antiretroviral treatment initiation as a priority for children aged more than 2 years and less than 5 years according to the 2015-revised WHO guidelines [6] and to the 2016-consolidated WHO guidelines [7]

^$^A 10% bilateral range (i.e., counts between 190 and 210 CD4 T cells/μl for the threshold at 200 CD4 T cells/μl; counts between 332 and 367 CD4 T cells/μl for the threshold at 350 CD4 T cells/μl; counts between 712 and 787 CD4 T cells/μl for the threshold at 750 CD4 T cells/μl; and counts 23.7 and 26.2 %CD4+ for the threshold at 25 %CD4+) was considered similar

^a^The sensitivity and specificity of CD4 T cell counting by the Muse™ Auto CD4/CD4% system to identify patients having less than (or more than) 200 CD4 T cells/µl and 350 CD4 T cells/µl, were calculated on the 150 available CD4 T cell count measurements from infants, children, adolescents and adults

The capability of the Muse™ Auto CD4/CD4% system to identify patients having less than (or more than) 200 CD4 T cell/µl was evaluated on the 150 available CD4 T cell count measurements. Taking into account a 10% bilateral range (i.e., counts between 190 and 210 CD4 T cell/µl were considered similar), the concordance between the Muse™ Auto CD4/CD4% system and BD FACSCount system was high (κ = 0.98; P < 0.01). The decision differed for only 1 study’s blood samples. Accordingly, the Muse™ Auto CD4/CD4% showed a sensitivity of 96.7% and a specificity of 100% to identify individuals with CD4 T cell counts below 200 cells/µl when compared with the results obtained by the BD FACSCount system.

The sensitivity and specificity of CD4 T cell counting in absolute number on the Muse™ Auto CD4/CD4% system to identify patients having less than (or more than) 350 CD4 T cell/µl was also evaluated on the 150 available CD4 T cell count measurements. Considering a 10% bilateral range (i.e., counts between 332 and 367 CD4 T cell/µl), the concordance between the Muse™ Auto CD4/CD4% system and BD FACSCount system was very high (κ = 1.0; P < 0.01). Accordingly, the Muse™ Auto CD4/CD4% system had a sensitivity of 100% and a specificity of 100% to identify individuals with CD4 T cell counts below 350 cells/µl when compared with the results obtained by the BD FACSCount system.

Considering the thresholds relevant in children, the Muse™ Auto CD4/CD4% system correctly identified all children having less than 750 CD4 T cell/µl, considering a 10% bilateral range (i.e., counts between 675 and 825 CD4 T cell/µl), but 5 children with less than 750 CD4 T cell/µl were misclassified, providing a low specificity of 83.9%; the Muse™ Auto CD4/CD4% system identified nearly all children correctly having less than  %CD4+ ≤25%, considering a 10% bilateral range (i.e., counts between 22.5 and 27.0 %CD4+), providing a sensitivity of 95.5%, without misdiagnosing (specificity of 100%). Finally, the concordance between Muse™ Auto CD4/CD4% and BD FACSCount systems to accurately classify patients according to the relevant pediatric thresholds was excellent in absolute counts as well as in percentages.

## Discussion

In the present study, we demonstrated that the Muse™ Auto CD4/CD4% system, when operated by a laboratory technician, performs acceptably compared to the BD FACSCount system when used as the reference method for CD4 T cell measurement expressed in absolute number as well as in percentage. The Muse™ Auto CD4/CD4% system flow cytometer results in absolute number and percentage gave perfect correlations with those obtained by the reference flow cytometer method. Through the Passing-Bablok regression analysis, the correlation was maintained over all the dynamic range of values in absolute number (until 2113 CD4 T cells/µl) as well as in percentage (until 59 %CD4+). CD4 T cell counting by Muse™ Auto CD4/CD4% system allowed to accurately identify the majority of individuals with CD4 T cell below 200 cells/µl and all individuals with CD4 T cell below 350, 750 cells/µl and 25 %CD4+ demonstrating the capacity of the Muse™ Auto CD4/CD4% system to accurately assess the major thresholds in absolute number (200, 350 and 750 cells/µl) or in percentage (25 %CD4+), used in clinical practice to initiate or follow ART in adults as children. The procedure was fast and needed only 30 min to be completed. The technique was found to be very easy to carry out and highly precise in terms of repeatability and reproducibility, with intra- and inter- run variabilities less than 15%, considered as acceptable for clinical use [10, 18]. The interperson variation, which could introduce further assay variation, was however not assessed in the present study. Furthermore, the number of included young children was low in our study, thus warranting further studies to fully confirm the interest of the system for CD4 T cell counting in children and infants. Taken together, these findings demonstrate that the Muse™ Auto CD4/CD4% system is a reliable, robust and cost-effective alternative flow cytometer for CD4 T lymphocyte enumeration to be used routinely for immunological monitoring according to the WHO recommendations in HIV-infected adults as well as children living in resource-constrained settings.

The WHO strongly recommends scientific reports of effective validation of newly introduced, affordable CD4 T cell measurement technologies, carried out in the field by several laboratories of different resource-poor countries, independently of manufacturers [31]. Therefore, the present independent validation of the Muse™ Auto CD4/CD4% system by two reference laboratories of Bangui well fulfilled the WHO recommendations for validation of CD4 T cell assays in resource-poor settings. The BD FACSCount system was used as reference system for CD4 T cell counting in the present evaluation. Indeed, the BD FACSCount is the oldest dedicated flow cytometer that has been extensively used since 1996 [32] and fully validated against reference flow cytometers in resource-limited settings [32–35].

The unique, patented micro-capillary flow cell technology used in the Muse™ Auto CD4/CD4% system eliminates the requirement for complicated sheath flow fluidics and enables absolute cell counts without the need for reference beads, making the system extremely compact, easy to maintain and simple to use. The scaling up of public ART programs globally has led to an increased demand for CD4 T cell count tests [36], especially to assess treatment eligibility. Furthermore, because ART may not be universally provided, the WHO defined priorities for ART initiation, all adults and adolescents with severe or advanced HIV clinical disease (WHO clinical stage 3 or 4) and individuals with CD4 count ≤350 cells/mm^3^, as well as all children from aged 3 to maximum 10 years old with severe or advanced HIV clinical disease (WHO clinical stage 3 or 4) and individuals with CD4% < 25% (if under 5 years old) or CD4 count ≤350 cells/mm^3^ (if under and up to 5 years old) [6, 7]. Finally, CD4 T counting remains currently an important biological marker for antiretroviral treatment initiation and monitoring, while HIV-1 RNA load is not sufficiently available in the field. The speed of implementation of CD4 T lymphocyte count facilities has been unrivalled in recent years in resource-limited countries and has met challenges with technology selection, laboratory infrastructure development, human resource training, cost-effectiveness, instrument maintenance and ensuring testing access and quality [14, 31, 37]. However, most conventional benchtop flow cytometer systems used for enumeration of CD4 T lymphocyte counts are relatively complex, costly, technically demanding and the instruments used, need constant maintenance. Costly maintenance, the need for well-trained laboratory staff and a cold chain to ship and store reagents, can limit the use of sophisticated analyzers. These systems are difficult to apply for routine use in most laboratories operating with poor facilities and in resource-limited settings. The Muse™ Auto CD4/CD4% system for CD4 T cell counting allows minimizing the sources of variations, is less expensive, is small, is simple to use and yet reproducible. Similarly, previous studies have yet reported that point-of-care non-flow cytometry-based CD4 T cell counting device, such as the PIMA™ CD4 from Alere (Alere, Jena, Germany) [38] or Partec CyFlow miniPOC from Partec (Partec GmbH, Munster, Germany) [39] may be interchangeable with the existing conventional CD4 T cell enumeration platforms [10]. All these more affordable CD4 dedicated flow cytometers introduced into the market are thought to ensure decentralization of the HIV-monitoring services [9, 10]. Indeed, point-of-care CD4 T cell counting technologies reduce the time and increase patient retention along the testing and treatment cascade compared to conventional laboratory-based testing [40], which are therefore considered to be useful tools to perform CD4 T cell counting for expedite result delivery.

## Conclusion

In resources-limited settings, CD4 T cell count remains an essential biological monitoring marker to diagnose and monitor antiretroviral treatment failure, because of frequent lack of HIV viral load availability. The Muse™ Auto CD4/CD4% system constitutes a promising system for performing single-platform, absolute and percent CD4 T lymphocyte counts with excellent reproducibility and should facilitate wider access to CD4 T cell enumeration for adults and children with HIV infection living in resource-constrained countries.

## Abbreviations

ART: antiretroviral treatment
*CNRISTTAR*: *Centre National de Référence des Infections Sexuellement Transmissibles et de la Thérapie Antirétrovirale*
CSVPR: *Comité Scientifique de la Validation des Protocoles et des Résultats de Recherche en Santé*
Cy™5: indodicarbocyanine
DP: dual platform
FACSS: *Faculté des Sciences de la Santé*
PE: phycoerythrin
SD: standard deviation
WHO: World Health Organization
